# Significance and implications of FDA approval of pembrolizumab for biomarker-defined disease

**DOI:** 10.1186/s40425-018-0342-x

**Published:** 2018-05-14

**Authors:** Michael M. Boyiadzis, John M. Kirkwood, John L. Marshall, Colin C. Pritchard, Nilofer S. Azad, James L. Gulley

**Affiliations:** 1 University of Pittsburgh, Pittsburgh, PA USA; 2 University of Pittsburgh Hillman Cancer Center, Pittsburgh, PA USA; 3 Lombardi Cancer Research Center, Washington, D. C., USA; 4 University of Washington, Seattle, WA USA; 5 Johns Hopkins University, Baltimore, MD USA; 6 Genitourinary Malignancies Branch, Center for Cancer Research, National Cancer Institute, National Institutes of Health, Bethesda, MD USA

**Keywords:** DNA mismatch repair, Microsatellite instability, Pembrolizumab, Biomarker, Immunotherapy

## Abstract

The U.S. Food and Drug Administration (FDA) recently approved pembrolizumab, an anti- programmed cell death protein 1 cancer immunotherapeutic, for use in advanced solid tumors in patients with the microsatellite-high/DNA mismatch repair-deficient biomarker. This is the first example of a tissue-agnostic FDA approval of a treatment based on a patient’s tumor biomarker status, rather than on tumor histology. Here we discuss key issues and implications arising from the biomarker-based disease classification implied by this historic approval.

## Background

The discovery and validation of immune checkpoints (signal-transducing pathways that modulate immune system activity) as therapeutic targets has transformed cancer immunotherapy [1]. The heavily studied immune system checkpoint, programmed cell death protein 1/programmed death-ligand 1 (PD-1/PD-L1) regulates T-cell function through the T-cell PD-1 receptor and the PD-L1 presented by targeted cells [1, 2]. Interactions between PD-1 and PD-L1 primarily inactivate CD28 signaling to suppress T cell activation [3]. Many approved immunotherapies inhibit PD-1/PD-L1 interactions in order to stimulate an immune response against cancer cells [2].

Pembrolizumab (KEYTRUDA®, Merck & Co., Inc., Kenilworth, NJ) is a humanized, mouse- derived anti-PD-1 antibody that promotes tumor-cell apoptosis by binding to T-cell PD-1 receptors and disrupting interaction with PD-L1 molecules on tumor cells [4, 5]. Pembrolizumab is approved for use in patients with melanoma, non-small cell lung cancer (NSCLC), head and neck squamous cell carcinoma, classical Hodgkin lymphoma, urothelial carcinoma, and gastric/gastroesophageal junction cancer [4].

There are multiple immunological factors that potentially contribute to pembrolizumab’s efficacy in subsets of patients with melanoma or NSCLC, among other cancers. Studies have noted that both melanoma and NSCLC display increased tumor immune infiltrate and PD-L1 expression [6–9]. Synergy exists between these two factors as well, as increased IFN-γ release by infiltrating immune cells can upregulate PD-L1 expression [10]. Additionally, melanoma and NSCLC are diseases that display increased tumor mutational rate and burden due to both environmental and behavioral factors. Increased tumor mutational burden can promote increased neoantigen expression, which promotes T cell expansion and recruitment [11, 12]. Thus, data supports a hypothesis that anti-PD-1 therapy may be more effective in tumors increased in mutational burden, but this has not been experimentally verified.

Cancer biomarkers are specific DNA/RNA/protein features that correlate with either risk of cancer progression (prognostic) or response to a specific therapy (predictive). Identification of cancer biomarkers has been a significant factor in recent changes in disease classification and therapy [13]. Two common predictive biomarkers, which are often found together, are tumor microsatellite instability (MSI) and DNA mismatch repair deficiency (dMMR). The dMMR biomarker indicates whether a tumor’s DNA mismatch repair (MMR) system is deficient (d), based on the mutation or methylation status of 4 genes: MLH1, MSH2, MSH6, and PMS2. These genes can be inactivated through hereditary (Lynch syndrome) or somatic (sporadic) mutation, or silenced through promoter methylation [14–16]. Tumors positive for the dMMR biomarker commonly accumulate mutations that expand and/or reduce specific repetitive DNA microsatellite sequences [15]. Mutational assessment of 5 diagnostic microsatellite sequences using a commercially available assay is considered the current standard for evaluating tumor microsatellite biomarker status. A tumor is designated MSI-high (MSI-H) if at least 2 of 5 microsatellites harbor mutations [17]. Other methods used to determine MMR status include immunohistochemistry for MMR gene products and next-generation sequencing (NGS) to assess microsatellites across the genome [18, 19].

MSI status is variable across cancer types. MSI-high (MSI-H) biomarker designation is common in endometrial cancers, but is rare in hepatic, biliary tract, and pancreatic cancers [20, 21]. Additionally, mutated microsatellite loci can vary between cancer types and tumor histology [21]. In an early phase I study of the anti-PD-1 agent nivolumab, one patient with dMMR-positive colorectal cancer (CRC) had a durable complete response [22]. In 2015, a small study first reported the potential efficacy of pembrolizumab in treating tumors with the MSI-H/dMMR biomarker. Researchers observed that patients with mismatch-deficient CRC who received pembrolizumab had 40% and 67% increases in objective response rate (ORR) and progression-free survival, respectively, compared to patients with mismatch-proficient tumors [23].

Combined data from disease-specific pembrolizumab clinical trials (KEYNOTE-016, KEYNOTE-164, KEYNOTE-012, KEYNOTE-028, and KEYNOTE-158) confirmed these findings, and on May 23, 2017 the U.S. Food and Drug Administration (FDA) granted accelerated approval for pembrolizumab in adult and pediatric patients with unresectable or metastatic solid tumors with positive MSI-H or dMMR biomarkers [4]. Full approval will require additional trials showing continued safety and efficacy. However, this marks the first tissue-agnostic approval of any drug and thus represents a paradigm shift, as oncologic diseases may now be classified by either tumor biomarker status or tumor histogenesis. Here we discuss the implications of this novel biomarker-based disease classification for cancer immunotherapy research and practice.

### The vision of biomarker-based treatment

This first FDA approval of a therapy based on tumor biomarker status aligns with the clinical vision of precision medicine—highly individualized, customizable health care that many believe is the future of cancer diagnosis and treatment. Oncologic precision medicine involves screening for, and selecting therapies based on, an individual’s tumor-specific biomarkers to enhance clinical outcomes and minimize adverse events. The use of imatinib for Philadelphia chromosome-positive patients with chronic myeloid leukemia (CML) is one of the earliest examples of a therapy designed to target a specific tumor biomarker. Imatinib, a tyrosine kinase inhibitor, was rationally designed to inhibit the breakpoint cluster region (BCR)-Abelson (ABL) fusion protein that arises in Philadelphia chromosome-positive patients (~ 90% of all patients with CML) [24]. Imatinib moved from initial human trials to FDA approval in CML settings in just 3 years, likely due to intelligent developmental program design [25]. Approval of pembrolizumab for treatment of MSI-H/dMMR-positive tumors continues this progression toward precision medicine.

Why was pembrolizumab the first anticancer agent to receive tissue-agnostic FDA approval? One likely factor was that the initial randomized pembrolizumab trials conducted across tumor types prioritized tissue collection. This allowed investigators to retrospectively test the tissue-agnostic hypothesis across a larger number of samples to strengthen their conclusions [7]. These data indicate the importance of acquiring tissue during clinical trials to support future hypothesis testing and sophisticated design of biomarker-based studies. Concerning this approval, retrospective data was verified through multiple prospective clinical trials (KEYNOTE-016 and KEYNOTE-164), emphasizing a design of initial randomized clinical trials validated with prospective, hypothesis testing analyses. The FDA has prioritized this type of clinical trial design, as evidenced by the request for a prospective trial to grant anti-PD-1 nivolumab an extended indication for treating MSI-H/dMMR tumors outside of the original colorectal cancer indication (CheckMate-142) [26]. It is also important to note that, in general, immunotherapies are rationally designed from a foundation of preclinical data, without which this groundbreaking tissue-agnostic FDA approval of an immunotherapeutic cancer drug might never have been achieved.

### MSI-H/dMMR as a biomarker for therapy selection

**Fig. 1:**
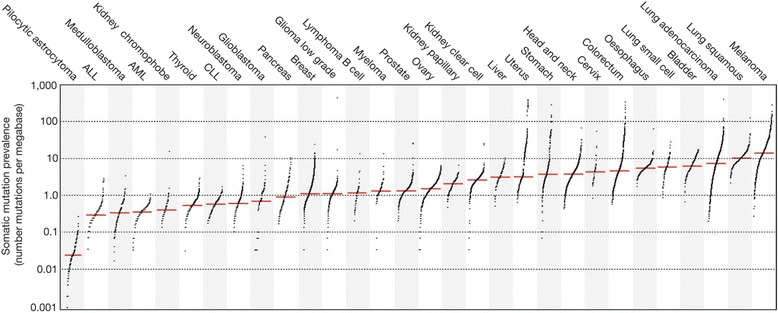
Prevalence of somatic mutations across respective cancer types. Each dot represents an individual sample and red horizontal lines represent the median number of mutations across samples. ALL, acute lymphoblastic leukemia; AML, acute myeloid leukemia; CLL, chronic lymphocytic leukemia. Adapted from 27

The MSI-H/dMMR biomarker has been used to guide prognosis for patients with stage II CRC, using tests such as Oncotype DX® [27, 28]. MSI-H/dMMR has also been used to predict the efficacy of chemotherapy for patients with CRC [29]. Although the presence of the MSI-H/dMMR biomarker varies across cancer types, clinical trials and pathophysiological studies indicate wide distribution of this biomarker across tumor types (e.g., uterine, gastric, CRC, liver, RCC) [21, 30]. This is especially apparent in cancers located in tissues exposed to a high burden of potential dietary mutagens, such as CRC and gastric cancers [15, 20, 21]. Additionally, the MSI-H/dMMR biomarker indicates tumor hypermutability, which can promote both immune system recognition and response to anti-PD-1 immunotherapies [31]. Cancers with the highest incidence of dMMR/MSI-H positivity, such as melanoma and NSCLC, also have increased prevalence of somatic mutations (Fig. 1) [32]. It is important to also consider how mutation may impact immunotherapy resistance. Anti-PD-1 resistance can arise through several mutation- derived mechanisms, including reduced interferon signaling through inactivation of JAK1 and JAK2, immune escape through HLA loss, as well as altered antigen presentation through loss of beta-2-microglobulin heterozygosity [33–35].

Using biomarker status to classify disease has a number of clinical implications. Perhaps most important, efficient and consistent biomarker testing methods will be required to ensure that patients are correctly selected for therapy. To date, no formal MSI-H/dMMR companion diagnostic accompanies approval of pembrolizumab for biomarker-based disease. Additionally, MSI-H/dMMR biomarker classification methods are evolving and can be assessed by various methods, including MSI mutational burden using PCR, MMR protein expression by immunohistochemistry, or using NGS to assess MSI across the genome [17–19]. The availability of multiple methods increases variability in determining patients’ tumor biomarker status [4, 17].

Developing standardized MSI-H/dMMR biomarker assessment protocols could reduce such variability. PCR methods, in particular, must account for tissue variability, as traditional methods were primarily validated for use in CRC and will have different sensitivity when applied in other cancers [16]. NGS methods that more thoroughly interrogate microsatellite loci across tumor types may provide the best approach for standardizing MSI classification [19]. Any developed technology will also require optimization in order to reduce false negatives and/or positives. Optimal tissue collection procedures, as well as reduced error rates in utilized sequencing technologies, will help in this regard. It will also be necessary to develop standardized recommendations to guide patient selection for biomarker assessment. Currently, guidelines from the National Comprehensive Cancer Network (NCCN) and the American Society of Clinical Oncology (ASCO) recommend that all patients with CRC be tested for MSI-H/dMMR biomarker status [36, 37]. Biomarker testing recommendations for patients with other tumor types will need to be developed over time. Biomarker testing will also become imperative for patients with metastatic disease who are approved to receive pembrolizumab [4]. Modifying and standardizing payment options for biomarker testing will also be critical, as variations in insurance coverage could reduce the number of patients who elect to receive biomarker-based therapy, even if they are potentially eligible for it.

### Drug development for biomarker-defined disease

Biomarker-based disease classification will require new approaches concerning drug development. Researchers will need to determine whether clinical trial design should be tissue- agnostic, investigating therapeutic efficacy against multiple tumor types according to biomarker status. One example of this strategy is the ongoing NCI-MATCH trial (Molecular Analysis for Therapy Choice) investigating the efficacy of a variety of therapies against solid tumors positive for a range of biomarkers, including sunitinib against tumors positive for cKIT mutation and afatinib against tumors positive for EFGR mutation [38]. Further preclinical biomarker research will be essential to the development of tissue-agnostic therapies. For example, we know that increased tumor PD-L1 expression is associated with PD-1-positive immune-cell infiltration.

Preclinical research may determine whether tumor PD-L1 status, activated T-cell infiltrate, or other immune checkpoint expressions are valuable adjuncts to MSI-H/dMMR status in predicting response to pembrolizumab [6, 7]. Standardized biomarker cutoffs will also need to be identified and incorporated across both drug development and clinical trial programs. PD-L1 positivity cutoff, for example, is variable across cancer types [39]. Drug development programs and clinical trials will require consistent biomarker cutoffs to ensure optimal therapeutic design and implementation.

Biomarker-based disease classification may affect the design of future combination therapies that target the PD-1/PD-L1 checkpoint. Combination immunotherapy may be more effective than monotherapy because of its potential to concurrently target multiple immune checkpoints [40]. Tissue-agnostic therapies may be excluded from traditional trials of combination therapies on the theory that it is not worthwhile to employ them towards tissue- specific cancers. The approval of pembrolizumab for MSI-H/dMMR-positive solid tumors provides a promising platform for future tissue-agnostic combination therapies. Approval of tissue-agnostic combination therapy, however, may require that regulatory bodies recognize that each drug within a combination therapy may not require prior tissue-agnostic approval as a monotherapy to eventually prove more effective in combination.

### Tissue-agnostic targeting of the PD-1/PD-L1 pathway

**Fig. 2:**
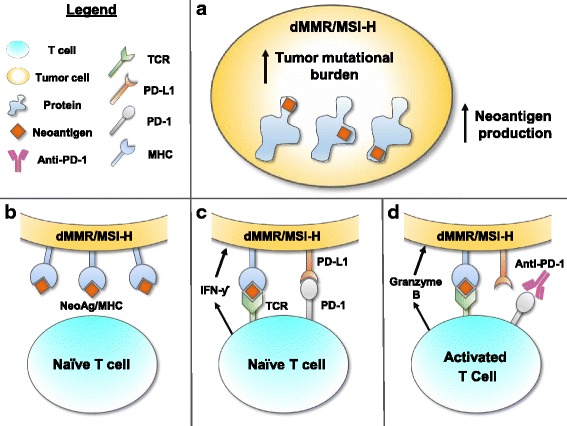
In patients with dMMR or MSI-H – positive tumors, multiple mutations accumulate and increase the likelihood of immunologically relevant neoantigens (**a**). Neoantigens are presented in the context of the MHC molecules on the tumor cells (**b**). T-cells specific for presented neoantigens can become activated initiating a series of molecular events including production and secretion of IFN-gamma by the T-cell (**c**). Among other things, this will cause up regulation of PD-L1 on the tumor cell which binds to PD-1 on the T-cell and sends a signal to inhibit activation (**c**). However, interruption of that negative signal (e.g., by an anti-PD-1 antibody) can reinvigorate the T-cell and promote anti-tumor activity (**d**)

The PD-1/PD-L1 checkpoint has been extensively studied, and PD-1 and PD-L1 inhibitors have induced encouraging clinical responses in patients with NSCLC, melanoma, Hodgkin lymphoma, urothelial carcinoma, RCC, and many other cancers [4, 5, 41–45]. The introduction of disease classification by tumor biomarker status makes it important to fully understand how dMMR affects the immune checkpoint. The precise mechanism of pembrolizumab’s effect on MSI-H/dMMR-positive tumors remains unclear. The most persuasive mechanistic hypothesis is that increased tumor mutational burden promotes neoantigen expression and T-cell expansion, which enhance the anti-PD-1 response [7]. Increased neoantigen expression by MSI-H/dMMR- positive tumors may also correlate with increased PD-L1 expression, which would promote PD-1-positive T-cell infiltration [6, 7]. While not confirmed experimentally, this hypothesis posits immune-cell infiltration and tumor mutational burden as key predictors of pembrolizumab efficacy in patients with MSI-H/dMMR-positive tumors (Fig. 2). Novel technologies that can quantify tumor-infiltrating CD8-positive T cells and/or mutational burden may help predict response to immunotherapy.

Clinical trials have established the optimal dose of pembrolizumab as 200 mg every 3 weeks for adults, and 2 mg/kg (up to 200 mg) every 3 weeks for children [4]. Pembrolizumab dosing for MSI-H/dMMR-positive solid tumors is the same [4]. Anti-PD-1 therapies have shown acceptable safety profiles to date, yet targeting the PD-1/PD-L1 checkpoint in MSI-H/dMMR-positive tumors exposes patients to the drug in many clinical contexts for which there are scant safety data. For example, approval of biomarker-based pembrolizumab included pediatric patients, despite the fact that children were excluded from all five dMMR/pembrolizumab clinical trials (median age: 55, 36% age 65 or older) [4]. In addition, since patients eligible for pembrolizumab will have failed prior treatment, a complete therapeutic history will need to be considered to ensure maximum efficacy and to limit adverse events [4]. It is possible, for example, that pembrolizumab will be less effective in patients who have received prior immunotherapies due to immune system cross-talk, since targeting one immune checkpoint through prior therapy may alter the activity of another, separate pathway. Cross-talk can also lead to T-cell exhaustion and reduce the efficacy of immunotherapy [2, 46].

### Clinical significance of biomarker-based disease classification

Biomarker-based disease classification may expand treatment options. Tissue-agnostic therapies will likely be administered to a larger pool of patients than tissue-specific therapies. However, relying on biomarker testing may limit therapy options. Limited access to specialized assays and tissue testing by laboratories certified by the Clinical Laboratory Improvement Amendments (CLIA) could deter the use of biomarker-based therapies, especially for smaller healthcare facilities. Increased access to CLIA-certified NGS-based platform testing, in conjunction with IHC readily performed across the country, could potentially mitigate this challenge.

Increased use of biomarker-based treatment could lead to more widespread use of cancer immunotherapy and other precision oncology treatments. Programs to educate patients about cancer biomarkers and the need for more tumor biomarker testing would be a boon to patients undergoing these novel therapies.

Of course, improved outcomes would be the best recommendation for biomarker-based therapies. It is noteworthy that in the phase III KEYNOTE-023 trial, patients with advanced PD-L1-positive NSCLC who received pembrolizumab scored higher on the European Organization for the Research and Treatment of Cancer (EORTC) core quality of life questionnaire than patients who underwent chemotherapy (6.9 [95% CI: 3.3–10.6] for pembrolizumab vs. − 0.9 [95% CI: 4.8–3.0] for chemotherapy) [47].

### Future directions for biomarker-based immunotherapies

Approval of pembrolizumab for biomarker-based disease increases the likelihood that other therapeutic agents and biomarkers will receive tissue-agnostic approval in the future. Nivolumab, another anti-PD-1 agent, appears well on its way to tissue-agnostic approval for dMMR-positive cancers. A recent study revealed a 24% ORR among patients with a range of non-CRC dMMR-positive cancers treated with nivolumab (*n* = 35; 95% CI: 11–41) [48]. Moreover, 31% (23/74) of patients with MSI-H/dMMR-positive metastatic CRC treated with nivolumab had investigator-assessed objective responses (95% CI: 21–43) [26]. Discovery of more biomarkers will also promote the development of tissue-agnostic therapies. Along with MSI-H/dMMR status, many clinical trials have verified the importance of tumor mutational burden and PD-L1 status in predicting response to treatment, providing additional impetus for developing tissue-agnostic therapies [39, 49]. The advancement of single-cell analytics, as well as broad-range biomarker assessment technologies, will also spur biomarker research and novel tissue-agnostic strategies.

## Conclusion

Advanced sequencing and diagnostic tools have given researchers and clinicians a new lens through which to view cancer. The “big picture” approach of classifying disease based on tumor location may be supplanted by the use of definitive biomarkers, which would naturally lead to treatments based on tumor biomarkers rather than histology-specific status. The FDA approval of pembrolizumab for advanced MSI-H/dMMR-positive solid tumors is a tipping point for disease reclassification based on tumor-specific factors, and propels oncology further toward the goal of precision medicine. Biomarker-based disease classification will allow clinicians to individualize treatment, which will enhance therapeutic response and reduce adverse events.

Beyond its use in the clinic, pembrolizumab can also serve as a blueprint for future therapies to gain approval for tissue-agnostic administration. To date, pembrolizumab is the only therapy to gain approval for the treatment of patients with MSI-H/dMMR-positive solid tumors, meaning that clinicians cannot simply substitute other checkpoint inhibitors in place. Each agent seeking a similar indication will require individualized clinical trial validation. Additionally, consistent biomarker assessment protocols and treatment regimens for a variety of patient populations must also be developed before new therapies can receive FDA approval. Finally, while researchers may identify new targetable biomarkers for pembrolizumab or any other therapy, these hypotheses must also be confirmed in both randomized and prospective clinical trials. This initial tissue-agnostic designation of any systemic anti-cancer therapy is a promising step for the field of oncology, but more work remains. The goal now is to continue improving clinical outcomes by validating and selecting optimal treatments based on a patient’s tumor biomarker profile.

## Abbreviations

ABL: Abelson
ALL: Acute lymphoblastic leukemia
AML: Acute myeloid leukemia
ASCO: American Society of Clinical Oncology
BCR: Breakpoint cluster region
CLIA: Clinical Laboratory Improvement Amendments
CLL: Chronic lymphocytic leukemia
CML: Chronic myeloid leukemia
CRC: Colorectal cancer
dMMR: DNA mismatch repair-deficient
EORTC: European Organization for the Research and Treatment of Cancer
FDA: U.S. Food and Drug Administration
IFN: Interferon
IHC: Immunohistochemistry
MHC: Major histocompatibility complex
MSI: Microsatellite instability
MSI-H: Microsatellite instability-high
NCCN: National Comprehensive Cancer Network
NGS: Next-generation sequencing
NSCLC: Non-Small cell lung cancer
ORR: Objective response rate
PCR: Polymerase chain reaction
PD-1: Programmed cell death-protein
PD-L1: Programmed death-ligand 1
RCC: Renal cell carcinoma
